# Supplementary material for: Hypoadrenocorticism in a Dog Following Recovery from Alpha-Amanitin Intoxication
Source: Vet Sci. 2023 Aug 3;10(8):500. doi: 10.3390/vetsci10080500 (PMC10459733; doi:10.3390/vetsci10080500)
Supplement: Supplementary file 1 [file vetsci-10-00500-s001.zip › vetsci-2491518-supplementary.pdf]

Table S1: Clinicopathologic parameters throughout the initial hospitalization (days 1-8), recheck examinations (day 15 & 50) and secondary hospitalization (days 24-26) of a dog that recovered from acute liver insufficiency due to amanita intoxication and subsequently developed hypoadrenocorticism.

	Reference interval	Day 1	Day 2	Day 3	Day 4	Day 5	Day 6	Day 7	Day 8	Day 15	Day 24	Day 25	Day 26	Day 34	Day 50
Hematocrit (%)	50-55	62.3	60.6				55.3				56.9				
Neutrophils (cells/uL)	3,000 – 10,500	8,847					8,204				6,261				
Lymphocytes (cells/uL)	1,200 – 4,000	2,181					1,875				3,010				
Eosinophils (cells/uL)	0 – 1,500	485					527				1,806				
Platelets (cells/uL)	150,000 – 400,000	70,000 (clumps seen)					126,000 (clumps seen)				34,000 (clumps seen)				
Sodium (mmol/L)	143-151	148			145	151					131		134	154	152
Potassium (mmol/L)	3.6-4.8	4.4			4.5	4.7					5.6		5.5	4.0	3.9
ALT (IU/L)	21 – 72	13,288	10,343	6,007	4,103	3,270	2,555	1,993	1,674	214	85		69	174	108
AST (IU/L)	20 – 49	1,830	467	167	135	110	73	69	73	33	84		50	40	26
ALP (IU/L)	14 – 91	1,508	1,783	1,567	1,387	1,502	1,509	1,465	1,550	893	447		356	968	353
GGT (IU/L)	0 – 5	17	18	13	8	11	20	5	7	<3	<3		<3	<3	<3
Albumin (g/dL)	3.4 – 4.3	3.3	3.3	3	2.6	2.8	2.9	3.1	3.3	3.7	3.3		3.1	3.3	3.7
Globulin (g/dL)	1.7 – 3.1	1.6	2.1	1.9	1.9	2.2	2.4	2.5	2.9	2.5	2.6		2.1	2.0	2.3
Cholesterol (mg/dL)	139 – 353	188	193	137	105	101	99	99	104	100	78		68	106	164

Total Bilirubin (mg/dL)	0.0 – 0.2	1.4	2.1	1.4	0.9	0.6	0.5	0.5	0.5	0.2	0.3		0.2	<0.2	<0.2
Glucose (mg/dL)	86 – 118	73	87	74	149	82	90	63	51	91	43		120	117	114
Creatinine (mg/dL)	0.8 – 1.5	0.8			0.7	0.9					0.8		0.6	0.6	
Blood Urea Nitrogen (mg/dL)	11 – 33	17	9	5	4	8	10	10	15	39	28		19	7	8
PT (secs)	7.0 – 9.3	21.4					9.9		8.2	8.3					
aPTT (secs)	10.4 – 12.9	21					15.7		13.9	15.2					
Fibrinogen (mg/dL)	109 – 311	<50					119		210	160					
D-dimers (ng/dL)	0 – 186	904					58		181	72					
Aldosterone (pmol/L)	14 – 957	42*													
Resting cortisol (ug/dL)	0.0 – 6.0	2.0 *										0.4			
Post ACTH stimulation cortisol (ug/dL)	0.0 – 6.0											0.4			

*Analytes measured retrospectively on a citrated plasma sample stored at -80°C.
